# Effect of pediatric mouthwashes on the color stability of dental restorations with composite resins. *In vitro* comparative study

**DOI:** 10.4317/jced.59959

**Published:** 2022-11-01

**Authors:** Lizbeth Martinez-Ccahuana, Evelyn Álvarez-Vidigal, Ernesto Arriola-Guillén, Denisse Aguilar-Gálvez

**Affiliations:** 1 Division of Pediatric Dentistry, School of Dentistry, Universidad Científica del Sur, Lima, Perú; 2 Division of Orthodontics, School of Dentistry, Universidad Científica del Sur, Lima, Perú

## Abstract

**Background:**

To evaluate *in vitro* the effect of three pediatric mouth rinses on the color stability of three different composite resins.

**Material and Methods:**

One hundred thirty-two disc-shaped samples (n = 132) were prepared, with 44 for each type of composite resin (n = 44), and the initial color values were measured with a spectrophotometer. Eleven (n= 11) samples were placed in each immersion substance, and 3 types of pediatric mouthwashes were used with distilled water as a control. The samples were placed in an incubator at 37°C for one hour (equivalent to 1 month of mouthwash use). and color measurement was performed. Color change values (ΔE*) were obtained and the results were statistically analyzed. Kruskal Wallis and Mann-Whitney U tests were used to compare the values between groups (*P*<0.05).

**Results:**

All composite resin samples showed color changes after immersion in the different solutions studied. The ΔE* showed that pediatric mouth rinses produced significant changes in color of the composites tested. The group of nanohybrid resins presented lower levels of change in color stability (ΔE * = 4.63), followed by bulk resins (ΔE * = 5.70) and, finally, nanoparticle resins presented greater effects in color stability. (ΔE *= 5.84).

**Conclusions:**

All the composite resin restorative materials used showed differences in color after immersion in the 3 mouthwashes and the distilled water analyzed, these changes should be taken into by dentists working with pediatric patients – o – by pediatric dentists.

** Key words:**Mouthwashes, color stability composite resins, bulk resins, surface roughness.

## Introduction

The increase in esthetic expectations in dentistry has led to the development of many types of esthetic restorative materials with different physical and chemical characteristics for clinical use (1,2).

Composite resins are among the materials frequently used in pediatric dentistry because of their excellent esthetic properties and ability to bond to the dental substrate (1). Contemporary composite resins have evolved over time with improvements in resin monomers, fillers and their agents, providing better physical, mechanical and esthetic properties. Therefore, composites can be recommended for dental restorations in the anterior and posterior sector (3).

Despite the significant development of esthetic materials in recent years, changes in the physical properties of restorations, specifically color stability, pose a challenge due to various factors in the oral cavity. Color stability of composite resins is affected by intrinsic and extrinsic factors. The consumption of various foods, drinks and poor oral hygiene can cause physicochemical reactions within the matrix in the superficial and deep layers of the restorative materials, producing changes in their properties. Likewise, the composition and size of the filler particles have a direct impact on surface conditions and susceptibility to extrinsic staining (4-6).

Mouthwashes are widely used for improving and stabilizing periodontal health due to their anti-inflammatory, antiseptic and analgesic properties. They are used to control the remineralization of teeth and the formation of biofilms, depending on the risk of caries and the development of periodontal disease (7-14). Although mouthwashes are widely recommended for chemical control of plaque, excessive use can damage dental restorations with composite resin due to the low pH and alcohol present in these solutions (3). The use of mouthwashes has been reported as having a relevant effect on the color stability of restorative materials (15).

The color stability of restorations in pediatric dentistry, especially for esthetic anterior restorations, is an important requirement that should be taken into account in the choice of restorative materials (16). Therefore, it is essential to choose mouthwashes that do not compromise the properties of the resins. Likewise, the materials and mouthwashes presenting the greatest benefits for the patient must be selected to achieve a good success rate in dental restorations. Studies in this respect are limited. Therefore, the present *in vitro* study evaluated the effect of three pediatric mouth rinses on the color stability of three different composite resins.

## Material and Methods

We performed a prospective, longitudinal, comparative, experimental, *in vitro* laboratory study.

-LED Lamp Wavelength Intensity Measurement

The measurement of the wavelength intensity of a LED lamp (Brand 3M ESPE Elipar™ DeepCure-L) was carried out using a radiometer (Brand WOODPECKER- LED Light Meter, model LM-1), programmed with a reading range of 1450 mW /cm2 according to this intensity the polymerization time of the composite resins tested – o – study samples tested.

-Preparation of the composite resins– o – the study samples

One hundred thirty-four blocks of composite resin were made according to ISO 4049, using polytetrafluoroethylene molds of 4 mm in diameter by 2 mm high (Brand 3M-ESPE, USA). The mold was placed on a glass slide with celluloid tape. A very thin layer of liquid Vaseline was applied with a microbrush on the inside of the mold and the composite resin was inserted (3M™ Filtek™ Z250 XT Nanohybrid Universal Restorative, shade A1 [3M-ESPE, USA], 3M™ Filtek™ Z350 XT Universal Nanoparticle Restorative, Enamel shade A1E [3M-ESPE, USA], Filtek™ Bulk Fill Restorative for Posteriors [3M-ESPE, USA]) with a single increment using a spatula, according to the manufacturer’s instructions.

Before polymerizing, celluloid tape was placed on top of a glass slide for objects with slight pressure on the surface to make it flat. The time of polymerization with the LED lamp (Brand 3M ESPE Elipar DeepCure-L, power of 1000-2000mW/cm2) followed the manufacturer’s specifications, depending on the type of composite resin used:

- 3M™ Filtek™ Z250 XT Nanohybrid Universal Restorative, shade A1 (3M-ESPE, USA): 10 seconds of light curing.

- 3M™ Filtek™ Z350 XT Nanoparticle Universal Restorative, Enamel shade A1E (3M-ESPE, USA): 10 seconds light-curing.

- 3M™ Filtek™ Bulk Fill Restorative for Posteriors (3M-ESPE, USA): 20 seconds of polymerization

After completing the polymerization process, the surfaces of the specimens were polished with polishing discs (3M® Sof-Lex™ Extra Thin Finishing and Polishing Discs) according to the manufacturer’s specifications. Surface debris was removed with distilled water (DW), and the blocks were stored in sterile 30 ml amber bottles according to the corresponding resin group. These bottles were filled with 20 ml of artificial saliva (1.5mM CaCl2, 0.9mM NaH2PO4, 0.15MKCL with pH 7.0) and placed in the incubator (Ivoclar Vivadent brand, Culture model) at 37ºC for 24 hours in order to reproduce the temperature of the oral cavity and thereby allow the restorative material to complete its polymerization process.

Thereafter, the samples were removed from the artificial saliva and the specimens were completely dried with a paper towel.

Shade determination was performed at baseline using the Vita Easyshade® Advance 4.0 spectrophotometer (VITA, Bad Säckingen, Germany).

The test samples of each group (Group A, Group B and Group C) were divided into three subgroups (n= 11) which were immersed in different mouthwashes as well as a control group immersed in DW:

Group A: In addition to a control group (DW), 44 cylindrical blocks of conventional nanohybrid composite resin - 3M™ Filtek™ Z250 XT nanohybrid Universal Restorative, shade A1 (3M-ESPE, USA), with a diameter of 4 mm and a height of 2 mm were prepared and divided into 3 subgroups for immersion in 3 pediatric mouthwashes and DW:

Subgroup a: Consisting of 11 blocks of composite resin submerged in pediatric mouthwash with cetylpyridine chloride + Xylitol + 113 ppm fluoride (Hello Kitty [HK] – unique Tuinies Brand flavor).

Subgroup b: Consisting of 11 blocks of composite resin submerged in pediatric mouthwash with 0.075% cetylpyridine chloride + 225 ppm fluoride (Colgate® Plax Kids [CPK]– Tutti frutti flavor).

Subgroup c: Consisting of 11 blocks of composite resin submerged in pediatric mouthwash with 0.05% cetylpyridine chloride + 226 ppm Fluoride (Dentito [DENT] - strawberry flavor).

Control Group d: Made up of 11 blocks of composite resin submerged in DW.

Group B: A control group and 44 cylindrical blocks of conventional Nanoparticle composite resin - 3M™ Filtek™ Z350 XT Nanoparticle Universal Restorative, Enamel shade A1E (3M-ESPE, USA) 4 mm in diameter by 2 mm high were prepared and divided into 3 subgroups for immersion in 3 pediatric mouthwashes and DW:

Subgroup e: Consisting of 11 blocks of composite resin submerged in pediatric mouthwash with cetylpyridine chloride + Xylitol + 113 ppm fluoride (Hello Kitty HK – unique Tuinies Brand flavor).

Subgroup f: Consisting of 11 blocks of composite resin submerged in pediatric mouthwash with 0.075% cetylpyridine chloride + 225 ppm fluoride (Colgate® Plax Kids CPK – Tutti frutti flavor).

Subgroup g: Consisting of 11 blocks of composite resin submerged in pediatric mouthwash with 0.05% cetylpyridine chloride + 226 ppm Fluoride (Dentito DENT- strawberry flavor)

Control Group h: Made up of 11 blocks of composite resin immersed in DW.

Group C: A control group and 44 cylindrical blocks of 3M™ Filtek™ Bulk Fill Restorative for Posterior bulk resin, 4 mm in diameter by 2 mm in height, were prepared and divided into subgroups which were immersed in 3 pediatric mouthwashes and DW.

Subgroup i: consisting of 11 bulk resin blocks submerged in pediatric mouthwash with cetylpyridine chloride + Xylitol + 113 ppm fluoride (Hello Kitty HK– Tuinies brand).

Subgroup j: Consisting of 11 bulk resin blocks submerged in pediatric mouthwash with 0.075% cetylpyridine chloride + 225 ppm fluoride (Colgate® Plax Kids CPK– Tutti frutti flavor).

Subgroup k: Consisting of 11 bulk resin blocks submerged in pediatric mouthwash with 0.05% cetylpyridine chloride + 226 ppm Fluoride (Dentito DENT- strawberry flavor).

Control Group l: Made up of 11 bulk resin blocks submerged in DW.

-Color measurement

The color determination process was carried out according to ISO 7491. The test was carried out twice:

First Measurement: The first measurement (basal) was made after 24 hours of immersion in artificial saliva.

Second Measurement: The second or final measurement was carried out after immersion of the resin samples in the different pediatric mouthwashes and DW as a control.

Color measurement was performed using the Vita Easyshade Advance 4.0 Spectrophotometer (VITA, Bad Säckingen, Germany). Each resin disk was handled using latex gloves and cotton tweezers to avoid contamination of the test sample. The reading tip of the digital spectrophotometer was placed directly on the sample, positioning the tip of the Easyshade on the upper face of the disc resting on the entire surface. The measurement button must be adjusted until it emits 3 sounds, which indicate the completion of the process. The data obtained was recorded in a data collection form. Color measurements in natural light in the same environment and on a black background were simultaneously collected to avoid bias of the results.

-Statistical analysis

Normal distribution of the results of all groups was determined by the Shapiro-Wilk test. The absence of normality was determined and Kruskal Wallis and Mann-Whitney U tests were performed to compare the values between the groups and determine the presence of significant differences. Significance was considered with a *P* value <0.05.

## Results

When analyzing the composite resins for each immersion solution, the nanohybrid resin (Filtek™ Z250 XT) presented the least change in color stability in all the immersion substances. The greatest effects of color stability were observed in the composite resin of nanoparticles (Filtek™ Z350 XT) with DW and DENT and in the bulk resin (Filtek™ Bulk Fill) with HK and CPK. When analyzing the magnitude of color change (ΔE *) according to the type of composite resin, the group of nanohybrid resins presented lower levels of change in color stability (ΔE * = 4.63), followed by the bulk resins (ΔE * = 5.70), with the nanoparticle resins presenting the greatest change in color stability (ΔE *= 5.84). Analysis of each group, showed that all the resins presented less change in color stability when immersed in the CPK pediatric mouthwash and greater change when immersed in the DENT pediatric mouthwash. When performing the analysis based on the immersion solutions, the CPK pediatric mouthwash had the least effect on the color stability of the composite resins, followed by DW, HK and DENT. On analysis of each group of immersion solutions, we found that distilled water and the 3 pediatric mouthwashes produced less color change in the Nanohybrid composite resins and the HK and CPK pediatric mouth rinses produced greater color change in the bulk resins. DW and the DENT pediatric mouthwash produced the greatest color changes in the nanoparticle composite resins. Table 1 shows a statistically significant interaction between all groups (*p* <0.001); that is, the effect of immersion in the pediatric mouthwashes significantly differed among the 3 types of composite resin. Table 2 shows that the ΔE * values following immersion in the HK, CPK and DW solutions significantly differed when comparing the nanohybrid composite resin with the nanoparticles and the bulk resins, while immersion in the DENT solution showed no significant differences (*p* > 0.05).

**Table 1:**
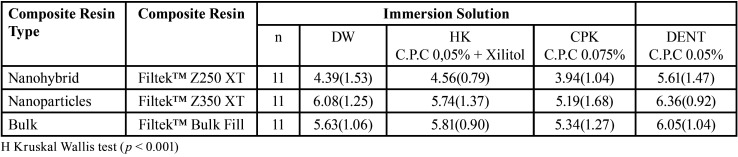
Mean value and standard deviation of the color stability (ΔE*) of the test materials in the different immersion solutions.

**Table 2:**
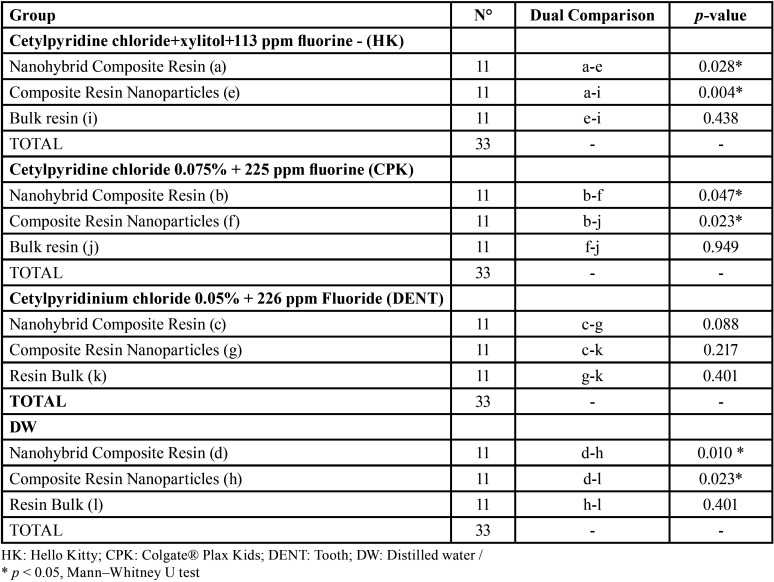
Comparison between the magnitude of color change (ΔE *) according to the immersion substances.

When performing the analysis according to the type of composite resin, Table 3 shows that the difference between the ΔE * values was statistically significant in the group of nanohybrid composite resins. There were no statistically significant differences in the ΔE * values with the nanoparticle composite resins and bulk resins immersed in the different mouthwash solutions, (*p* > 0.05).

**Table 3:**
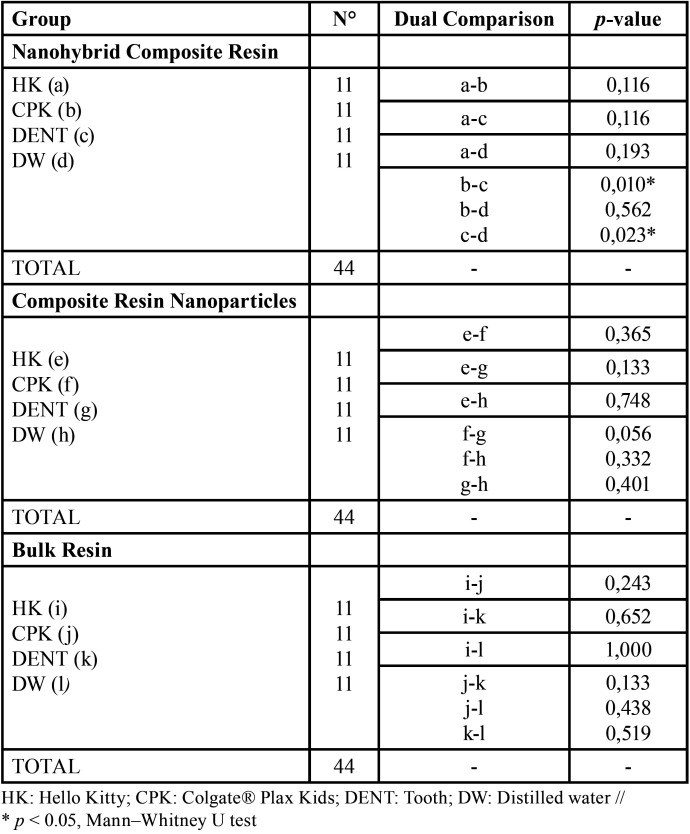
Comparison between the magnitude of color change (ΔE *) values according to the type of composite resin.

## Discussion

The present study analyzed the effect of 3 pediatric mouth rinses on the color stability of 3 different composite resins. All the restoration materials studied showed changes in color after immersion in the 4 mouthwash substances, very similar to the results of Celik *et al*. (4) and Nasim, Neelakantan, Sujeer (6).

Composite resins that contain fewer fillers are more prone to discoloration (5,6). In our study, it was found that composite resins with lower loads were more prone to color alterations. Bulk resin showed the lowest load with 76.5%, followed by nanoparticle resins with 78.5% while the nanohybrid resins with a high inorganic filler load presented a load of 81.8%. These latter resins with the highest load showed better color stability compared to the other 2 resins suggesting that the bulk and nanoparticle resins could have absorbed more water in the filler-matrix interface.

The discoloration of materials is also related to the roughness of their surface (5-7). An increase in the size of the restorative material particles induces greater absorption of water through the polymer chains, affecting the bonds between the matrix and the filler particles and producing an irregular surface during polishing treatment (8,9). In our study, the polishing process was carried out in a standard way and according to the manufacturer’s specifications for each composite resin used.

The present study also found that nanohybrid resins with larger particle size presented greater color stability; that is, they were less affected by the pediatric mouthwashes, while nanoparticle resins with a smaller particle size (average 0.6-10 microns) showed greater changes in color stability.

The significant color change in the Z350 resins after immersion in the mouthrinses studied was attributed to the presence of porosities in the aggregate filler particles and the porosity of the glass fillers, coinciding with the study by Nasim, Neelakantan, Sujeer (6). Several studies have reported that the type of material plays an important role in stain resistance, (4-10) the matrix and the size of the particles.

Likewise, our study showed that Bulk resins showed greater color change compared to nanohybrid resins, likely attributed to their content of aliphatic amines such as dimethylamine. These findings are similar to those of Nasim *et al*. (6) who showed that the microfilled resin (Heliomolar) presented greater discoloration than the microhybrid resin, which they attributed to the high content of aliphatic amines in the composition.

The water content of the mouthwashes can affect the change in color and microhardness, Therefore, the DW control group produced the same effects as the mouthwashes (11). In our study, the results showed color alterations in all the materials immersed in DW and statistically significant differences were found when comparing pairs (nanohybrid resin, nanoparticle resins and bulk resins).

When evaluating the surface degradation (mass and roughness) of nanohybrid composite resins subjected to contact with mouthwashes, Casanova *et al*. (12) observed that Resin Filtek Z250 XT (3M ESPE) presented less roughness than the Grandio resin (VOCO). In our study, the same nanohybrid Filtek Z250 XT (3M ESPE) resin was used and presented less color change after immersion in pediatric mouthwashes. Taking into account the surface roughness results of the aforementioned study, it can be suggested that the smaller the change in the surface roughness of the nanohybrid resin, the smaller the probability of its color change.

Several studies (17-19) have evaluated the surface hardness of nanotechnology resins, according to the time of polishing and observed that the nanohybrid resin presented greater surface hardness than the nanofilled resin for immediate polishing as well as for polishing after 24 hours; Therefore, we assume that the lesser color change presented by the nanohybrid resin in our study is also due to the presence of greater surface hardness, thereby producing less degradation of the filler and less susceptibility to color change.

## Conclusions

-In vitro evaluation of the effect of pediatric mouthwashes on the color stability of composite resin restorations showed color changes in all the restorative materials after immersion in the solutions studied.

-The pediatric mouthwash composed of Cetylpyridine Chloride at 0.075% + 225 ppm of fluoride caused less color change in the composite resins while the mouthwash producing the greatest color change was Cetylpyridine Chloride at 0.05% + 226 ppm of fluoride.

-The nanohybrid composite resin presented greater color stability with the different immersion substances, while the nanoparticulate composite resin presented lower color stability.

-Resin color stability is related to the composition of the matrix, as well as the composition and size of the filler particles and the volume percentage of filler load. Despite the advances in technology providing resins with better properties, there continue to be problems with color stability.
